# FUTURA STUDY-Evaluation of efficacy and safety of rupatadine fumarate in the treatment of persistent allergic rhinitis

**DOI:** 10.1016/S1808-8694(15)30517-6

**Published:** 2015-10-18

**Authors:** Olavo de Godoy Mion, Regis A. Campos, Martti Antila, Priscila Bogar Rapoport, Nelson Rosario, João Ferreira de Mello, Roberto Eustáquio Santos Guimarães, Marcos Mocellin, Giovanni Di Gesu, Dirceu Solé, Luc Wexler, João Ferreira de Mello, Fábio Morato Castro, Maria Letícia Chavarria

**Affiliations:** 1 PhD; Professor of Otorhinolaryngology - USP; 2 Post-Doctoral Research Fellow – Allergy Department - Yale University School of Medicine, USA, Adjunct Professor – Medical School – Federal University of Bahia; 3 MD. Allergist. ASBAI Executive Director - São Paulo Allergy Department ENT and Ophthalmology Hospital - Sorocaba; 4 Full Professor of Otorhinolaryngology – Medical School of the ABC – São Paulo; 5 PhD, Full Professor of Pediatrics – Federal University of Paraná; 6 Associate Professor – University of São Paulo Medical School; 7 Associate Professor – Department of Otorhinolaryngology and Ophthalmology of the Medical School of the Federal University of Minas Gerais- UFMG; 8 Full Professor and Head of the ENT Department – Federal University of Paraná; 9 Specialist in Allergy and Immunopathology; Attending Physician – Allergy Department - Santa Casa de Porto Alegre Hospital and Santo Antônio Children Hospital – Porto Alegre (RS); 10 Full Professor of Allergy, Immunology and Rheumatology – Department of Pediatrics – Paulista School of Medicine at the Federal University of São Paulo - UNIFESP-EPM; 11 Full Professor of Otorhinolaryngology - UNIFESP; 12 Executive Director, Head of the Allergy Department – State Public Servant Hospital - São Paulo; 13 Associate Professor – University of São Paulo Medical School; 14 Adjunct Professor-UFG, MSc in Immunology - USP and specialist in Allergy and Clinical Immunology /ASBAI-CFM. University of São Paulo – Main Center; Federal University of Bahia; Allergy Department – ENT and Ophthalmology Hospital -; University of the São Paulo ABC; Federal University of Paraná; Federal University of Minas Gerais; Federal University of São Paulo State Public Servant Hospital - São Paulo; Santa Casa de Porto Alegre and Children Hospital - Santo Antônio - Porto Alegre (RS)

**Keywords:** perennial allergic rhinitis, therapy

## Abstract

Allergic rhinitis affects 10-30% of the population, negatively impacting one's quality of life and productivity. It has been associated with sinusitis, otitis media, sleep disorders, and asthma. Rupatadine is a second generation antihistamine with increased affinity to histamine receptor H1; it is also a potent PAF (platelet-activating factor) antagonist. It starts acting quite quickly, offers long lasting effect, and reduces the chronic effects of rhinitis.

**Aim:**

this study aims to assess the efficacy and safety of rupatadine in the treatment of persistent allergic rhinitis.

**Materials and method:**

this is a multi-centric open prospective study. This study included 241 patients from 13 centers in Brazil and was held between October of 2004 and August of 2005. Signs and symptoms of rhinitis and tolerance to medication were analyzed after one and two weeks of treatment.

**Results:**

reduction on general scores from 8.65 to 3.21 on week 2 (p<0.001). All signs and symptoms improved significantly in the first day of treatment (p<0.001), except for nasal congestion and secretion, which improved from the second day of treatment (p<0.001). Adverse events occurred in 19.9% of the cases, 27.7% on week 1.

**Conclusion:**

rupatadine effectively controls persistent allergic rhinitis; it is safe and presents low incidence of side effects.

## INTRODUCTION

Persistent allergic rhinitis is a disease of variable prevalence that affects 10-25% of the world's population1, getting to as high as 30% in some populations^2^,^3^. The last 20 years saw a progressive increase in the global prevalence of the disease, resulting in greater health care expenditure^4^. Persistent allergic rhinitis is not a fatal disease, but it brings about significant clinical complications as it impairs patient quality of life, productivity and social life^1^. Rhinitis is an important predisposing factor for other conditions such as sinusitis and otitis media^3^; it may also induce or worsen sleep disorders and impact cranial-facial development in children^4^, apart from being intrinsically associated with asthma.

Allergic rhinitis is an immune-mediated disease in which mast cells and basophils initially respond to the triggering stimulus by inducing degranulation and release of inflammation mediators such as histamine after an antigen antibody reaction with IgE. Further on other cytokines are released, such as IL-4, IL-5, IL-6, IL-8, TNF-alpha, GM-CSF, RANTES, eotaxins, and PAF (platelet-activating factor) to maintain the inflammatory process.^5^, ^6^, ^7^, ^8^, ^9^

From the clinical standpoint, allergic rhinitis can be categorized into four subgroups (mild intermittent, moderate/severe intermittent, mild persistent, moderate/severe persistent)^1^. The intermittent manifestations can interrupt the triggered inflammatory process and return to the baseline status, but the persistent forms keep the pro-inflammatory mechanisms active with the participation of T-cells in a response mediated by lymphocyte Th210. In these cases, therapy based exclusively on antihistamines may not be enough, as other sites of the inflammatory process may require attention.

Rupatadine is a second generation antihistamine and a potent PAF antagonist with high affinity for receptor H111. These characteristics grant it fast action, long duration of effects, and reduction of the effects that sustain inflammation, traits that could turn it into the ideal medication for patients with persistent allergic rhinitis and active inflammatory process.

Studies on antihistamines are usually conducted in countries where the presence of seasonal rhinitis is quite significant. However, as Brazil is a tropical country in which the seasons are not so well-defined, seasonal rhinitis is not as prevalent as persistent rhinitis and rhinitis caused by occupational exposure. This environmental trait may imply in pathophysiological differences, with predominant Th2 inflammatory response types mediated by cytokines such as eotaxins, RANTES and PAF^9^,^12^. Therefore, the usual antihistamines may not be as effective here as found in studies carried out in Europe and the United States. On the other hand, antihistamines such as rupatadine that present both antihistamine and anti-PAF effect may be quite effective in populations with a similar profile to that of Brazil. Rupatadine is a new antihistamine and, for that reason, has not been targeted by a significant number of studies. This is the first study done in Brazil with this new antihistamine. It is relevant that multicentric studies be carried out in various places with different populations, so that efficacy and tolerability are tested in specific populations. This study aims to assess clinical efficacy and safety of rupatadine in a 2-week treatment program designed for patients with persistent allergic rhinitis.

## MATERIALS AND METHOD

### Studied population

Sample size was calculated taking into account an expected reduction of rhinitis signs and symptoms in treated patients ranging between 45% and 68% as seen in previous trials, and using the formula for population proportion of unknown size. Seventy-two patients had to be included in the study to allow for a confidence interval of 95% and a variation of 11.5% in population proportion.

Enrollment criteria: patients had to be above 12 years of age and diagnosed with moderate to severe persistent allergic rhinitis (according to the ARIA criteria) for at least one year, presenting symptoms for more than four days a week for at least four weeks, impacted quality and quantity of sleep, and interference upon daily life activities. Patients were enrolled in the study only after they or their guardians signed a Free Informed Consent Term.

Exclusion criteria: pregnancy, nursing patients, patients taking other medications for the treatment of rhinitis (oral steroids for one month, topical steroids for one week, leukotriene receptor antagonists (LTRA) for one week, other antihistamines for 15 days), patients with acute airway disorders such as upper airway infections or non-allergic eosinophilic rhinitis, drug-induced rhinitis or patients with other conditions that could potentially introduce confusion in result interpretation, excessively deviated septum, nasal polyps, any nasal malformation, or who present current evidences of clinically significant diseases of a hematopoietic, cardiovascular, hepatic, renal, neurologic, psychiatric, autoimmune, or respiratory nature or severe atopic dermatitis.

### Study design

This is a multicentric open prospective study carried out to assess the clinical response of patients with moderate to severe perennial allergic rhinitis after two weeks of treatment with rupatadine fumarate. Patients were selected from 13 centers in Brazil between October of 2004 and August of 2005, covering spring, summer, fall and winter, i.e., all seasons of the year. Some patients were assessed in southern states of Brazil, located in temperate latitudes where seasons are better defined. Patients had to provide written consent to participate in the study, meet enrollment criteria and have no exclusion criteria before they were assessed for their baseline parameters to allow the analysis of medication efficacy and safety; they were then given a diary of symptoms and the medication itself. Patients were reassessed one week and two weeks into the protocol. All parameters were looked into during the two follow-up visits so as to assess drug efficacy and safety. Patients were advised to take the medication in the morning as they woke up and to write down the symptoms they had every day in the morning as they got up (before they took the medication) and at night before they went to bed.

The study was approved by the Ethics Committees of all involved institutions under the principles of the Declaration of Helsinki.

### Efficacy assessment

Patients were asked to rate the rhinitis symptoms they experienced (nasal obstruction, nasal pruritus, sneezing, and nasal dripping) on a daily basis in a scale ranging from 0 (no symptoms) to 3 (hard-to-tolerate symptoms leading to adverse impact on everyday life and/or sleep). General symptoms (nasal obstruction, nasal pruritus, sneezing, and nasal dripping) and signs (turbinate coloration, presence of nasal discharge, and turbinate edema intensity) were also analyzed one and two weeks into treatment during medical visits. Assessment of signs was performed through observation during ENT physical examination done using a nasal speculum, and scores were assigned from 0 (normal) to 3 (anemic/bluish turbinate; profuse nasal discharge and drainage; one or two congested nasal fossae due to turbinate edema). Each sign was assessed separately, based on the sign and symptom score protocol described above^3^. Patient general impressions over clinical efficacy were also considered in the form of the diary they filled up in the morning and at night.

### Safety assessment

Safety assessment was carried out through a descriptive analysis of the adverse events observed during the study with possible, probable or defined causal relationships defined for the studied medication, according to the terminology for adverse reactions from the World Health Organization (WHO-ART); causality was assessed based on the Kasch-Lasagna algorithm. Systemic safety tests were carried out (CBC, creatinine, total bilirubin, AST and ALT). Researchers' impressions were also analyzed for patient tolerability based on the score given on the last visit two weeks into treatment.

### Statistical analysis

The data sets are presented in the form of mean values and standard deviations, followed by a descriptive analysis. Quantitative parameters were analyzed in terms of variance for repeated numbers, or the Friedman non-parametric test, adopting a significance level of 0.05 (p<0.05). Demographic results and clinical characteristics are analyzed for the population intended for treatment (PIT), efficacy is analyzed for the population following the protocol (PP), and safety is analyzed for the population intended for treatment (PIT).

## RESULTS

### Demographic data

**Figure 1:**
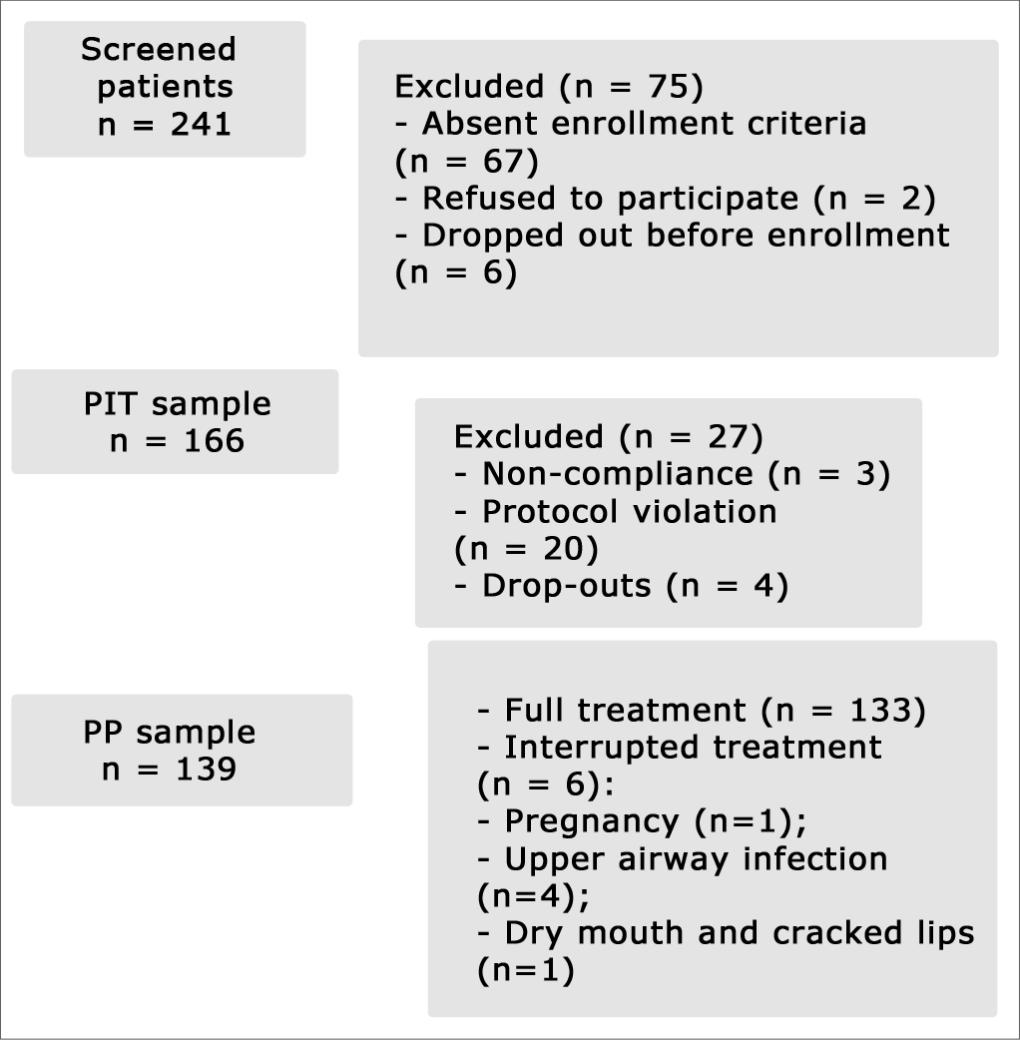
Flowchart - patient enrollment and follow-up.

A total of 241 patients applied for enrollment in the study. Seventy-five of them were excluded. Out of the 166 enrolled patients (PIT sample), 27 were excluded due to non-compliance, dropping out, or protocol violation (Fig. 1); 139 patients remained in the study (PP sample). Six of the remaining 139 interrupted the treatment due to pregnancy, upper airway infection, and xerostomia (Fig. 1).

Table 1 shows the overall distribution of patient demographic traits at the time of enrollment and patient clinical characteristics.

**Table 1:** Baseline clinical and demographic characteristics (before enrollment) PIT sample

Rupatadine	n = 166
Gender - n (%)	
Male	61 (36.7)
Female	105 (63.3)
Age (years) - mean ± SD	26.4 ± 11.2
Ethnicity - n (%)	
White	109 (65.7)
Black	22 (13.2)
Brown	33 (19.9)
Asian	2 (1.2)
Time with allergic rhinitis (years) - mean ± SD	11.3 ± 8.7
Allergic rhinitis intensity total score - mean ± SD	9.2 ± 1.6
Smoking - n (%)	7 (4.2)
Physical activity (at least twice a week) - n (%)	53 (31.9)
Previous anti-allergy medication * - n (%)	105 (63.3)
Concurrent diseases - n (%)	63 (38.0)
Weight (kg) - mean ± SD	61.9 ± 14.0
Height (cm) - mean ± SD	163.7 ± 9.7
Turbinate coloration - n (%)	
Reddish / pale pink	38 (22.9)
Red / pale	105 (63.3)
Anemic / bluish	23 (13.8)
Absent turbinate edema - n (%)	2 (1.2)
Absent turbinate discharge - n (%)	9 (5.4)

* -Previous use of the following anti-allergic drugs was reported: 75 (45.2%) antihistamines, 81 (48.8%) steroids, 3 (1.8%) adrenergic agents, 12 (7.2%) combined steroids and adrenergic agents, 1 (0.6%) chromone, and 2 (1.2%) immunotherapy.

### Overall efficacy

Efficacy assessment comprised the separate and combined evaluation of each sign and symptom of allergic rhinitis observed after one and two weeks of treatment, as well as the time it took for patients to improve from symptoms as recorded in the diary.

**Graph 1:**
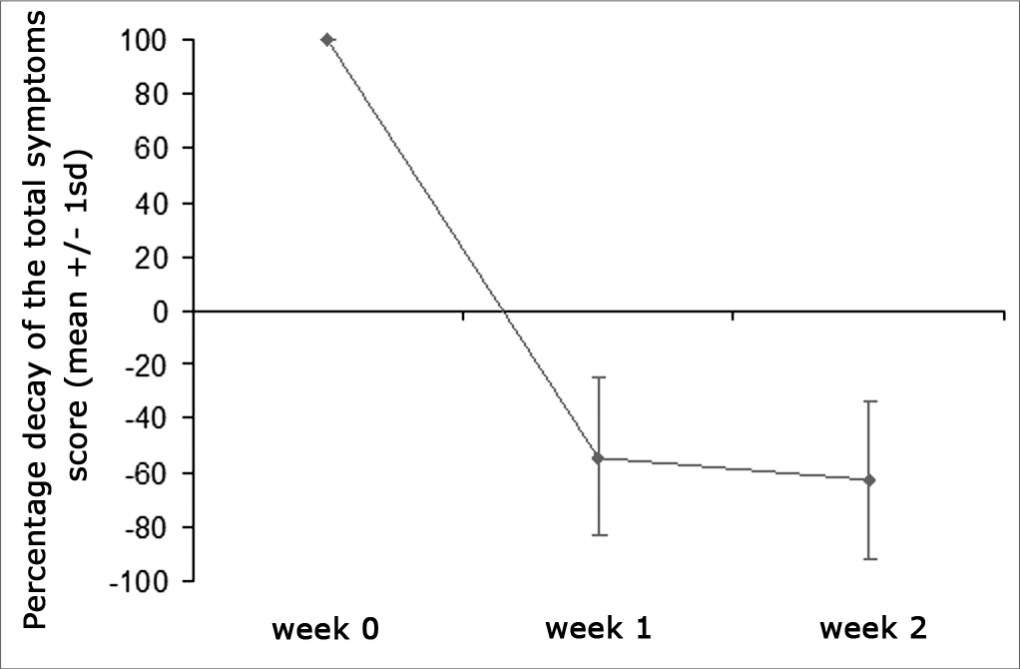
Percent decrease on overall symptom scores throughout the study. Mean values + standard deviation. Pre-treatment* p<0.05 in relation to pre-treatment, § p<0.05 in relation to week 1.

Overall symptom scores were significantly reduced from 8.65 to 3.88 after one week of treatment, and to a further 3.21 after two weeks of therapy (p<0.001) (Graph 1). All analyzed symptoms improved significantly after one week; only nasal obstruction did not show incremental improvement between the first and second week of treatment. Clinical nasal signs were significantly reduced after one week of treatment (p<0.001), but additional improvements were not observed after the second week of treatment (Table 2).

**Table 2:** Two-week evolution of signs and symptoms

Evaluation - mean + SD	Treatment period		
	Week 0 (pre-treatment)	Week 1	Week 2
Nasal pruritus	2.19 + 0.7	0.83 + 0.8 *	0.68 + 0.8 *#
Sneezing	2.22 + 0.7	0.66 + 0.8 *	0.50 + 0.7 *#
Nasal dripping	2.18 + 0.7	1.21 + 0.9 *	0.96 + 0.9 *#
Nasal obstruction	2.07 + 0.7	1.18 + 0.9 *	1.08 + 0.8 *
Total score	8.65 + 1.5	3.88 + 2.5 *	3.21 + 2.5 *#
Turbinate coloration	1.89 + 0.6	1.42 + 0.7 *	1.21 + 0.7 *
Turbinate edema	1.93 + 0.6	1.45 + 0.7 *	1.32 + 0.7 *
Turbinate discharge	1.62 + 0.6	0.88 + 0.7 *	0.71 + 0.7 *#

* p<0.001 in relation to week 0 (pre-treatment);

# p<0.001 in relation to the first week of treatment.

**Graph 2:**
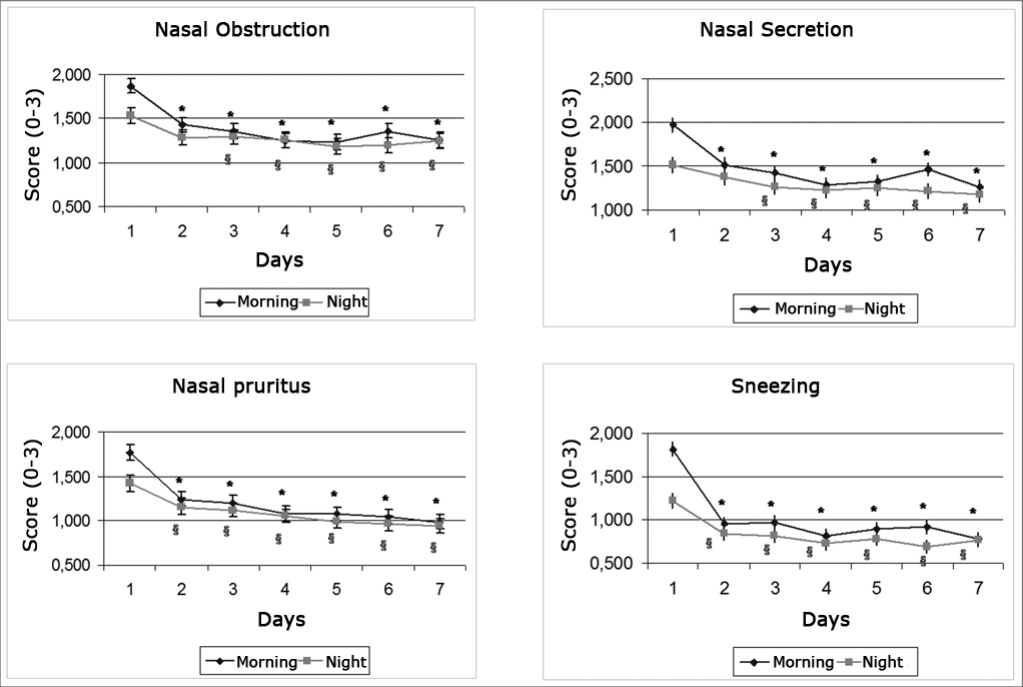
Daily evolution of nasal symptoms within the first seven days of treatment. * - p < 0.001 when compared to morning of day 1, § - p < 0.001 when compared to afternoon of day 1

Patients reported that all symptoms improved significantly right after the first day of treatment both in the morning and at night (p<0.001), except for nasal obstruction and discharge; these two only improved at night starting on the second day of treatment (p<0.001) (Graph 2)

### Safety

The study revealed an overall 19.9% prevalence of adverse events; in the first week of treatment this same prevalence rate amounted to 27.7%. The most relevant adverse events observed were sleepiness, increased appetite (3.1%), dry mouth (2.5%), and headache (3.7%). All such events had their prevalence reduced by the end of the study (Table 3).

**Table 3:** Adverse reactions observed during the study

Adverse reactions	Week 1 (n equals; 162) # patients (%)	Week 2 (n equals; 161)
Increased appetite	4 (2.5 %)	5 (3.1%)
Increased indirect bilirubin	0	2 (1.2%)
Increased total bilirubin	0	1 (0.6%)
Bitter mouth	1 (0.6%)	0
Dry mouth	4 (2.5%)	2 (1.2%)
Dry mouth and cracked lips	0	1 (0.6%)
Headache	6 (3.7%)	3 (1.9%)
Constipated bowel	0	1 (0.6%)
Impotence	0	1 (0.6%)
Nausea	1 (0.6%)	0
Epigastric burning sensation	1 (0.6%)	0
Skin rash	1 (0.6%)	0
Sleepiness	31 (19.1%)	23 (14.3%)
Dizziness	2 (1.2%)	1 (0.6%)
Vomiting	1 (0.6%)	0

Eighteen (11.1%) patients suffered from sleepiness throughout the entire study; 13 (8.0%) patients had sleepiness only during the first week; and only 5 (3.1%) patients had no sleepiness in the first week only to present it in the second week of treatment. Only three (1.85%) patients had severe sleepiness to the point of compromising their daily activity, and 14 (8.64%) had moderate sleepiness episodes. All remaining adverse events reported as mild and not commented above are shown on Table 3.

## DISCUSSION

This study has proven the efficacy of rupatadine in treating patients with persistent allergic rhinitis, reducing nasal symptoms, improving signs secondary to mucosal inflammation with sustained and even improving results after two weeks of treatment. The study has also shown that the rate of adverse events is relatively low and of little clinical significance.

Studies on antihistamines are usually conducted in countries where the presence of seasonal rhinitis is quite significant. However, as Brazil is a tropical country in which the seasons are not so well-defined, seasonal rhinitis is not as prevalent as persistent rhinitis and rhinitis caused by occupational exposure. This environmental trait may imply in pathophysiological differences, with predominant Th2 inflammatory response types mediated by cytokines such as eotaxins, RANTES and PAF^9^,^12^. Therefore, the usual antihistamines may not be as effective here as found in studies carried out in Europe and the United States. On the other hand, antihistamines such as rupatadine that present both antihistamine and anti-PAF effect may be quite effective in populations with a similar profile to that of Brazil^13^. Therefore, the main purpose of this study was to assess the degree of efficacy of an antihistamine with anti-PAF effect without however comparing it against other therapies. For this reason, the study was designed to assess response along time (two weeks) without using a control group.

From the demographic standpoint, the studied population was mainly composed of Young patients with a slight predominance of females (63.3% against 36.7% of males), with different ethnic backgrounds and moderate persistent rhinitis (symptom score of 9.2 out of 12). This patient profile matches the population distribution observed in the large Brazilian centers at which this study was carried out ^14^.

Classically, ordinary antihistamines do not offer good clinical response in controlling signs and symptoms such as nasal obstruction, edema and hyperemia that occur secondary to persistent inflammation^15^. This study showed that, contrary to what has been observed for other antihistamines^16^, rupatadine is effective in controlling all symptoms associated with rhinitis and provides for marked reduction on overall symptom scores. Improvements were statistically significant for nasal obstruction, even at night, as reported by the patients. This shows that rupatadine can effectively manage the persistent symptoms of allergic rhinitis even in cases where symptoms such as edema and nasal congestion persist for several months. Allergic nasal obstruction is caused by mucosal inflammation and edema, with engorged sinus capacitance vessels, rhinorrhea and increased mucus production, one of the symptoms more closely related to chronic nasal mucosa inflammation. Antihistamines are usually considered to be less effective in treating nasal obstruction than other symptoms of allergic rhinitis. Some other newer antihistamines have been associated with nasal obstruction relief16. Other studies on rupatadine have reported similar results, as this is a potent histamine receptor antagonist with good in-vitro anti-inflammatory effect^17^,^18^.

This study showed that rupatadine offers effective response in the treatment of persistent allergic rhinitis added by with two other important factors: fast response onset - observed right between the first and second day of treatment - and sustained improvements throughout the two-week period.

Other antihistamines do not offer consistent response against the main symptoms of allergic rhinitis. Therefore, patients often respond only partially to treatment and require additional drugs or even more time to control their symptoms^19^. The better management of signs and symptoms associated with persistent nasal inflammation offered by rupatadine may stem from the fact that rupatadine is a potent PAF antagonist, as shown in other studies^20^,^21^. However, the design of this study never meant to assess the role of rupatadine as a PAF antagonist; more studies should be conducted to assess the relevance of such trait for the treatment of human beings.

This study showed that there was a slight improvement on the symptom scores between the first and second week of treatment, and that treatment response was sustained by the end of treatment. Although this was only a two-week long study, sharper responses in symptom reduction were observed within the first week of therapy, as the medication acted quite rapidly and achieved close to full effect within seven days. On the second part of the study symptom mitigation was not as marked, as they had been dramatically reduced by then.

Prevalence of adverse events was generally low; the most relevant ones were related to sleepiness, although this is a relatively difficult factor to be analyzed. Sleepiness is a symptom of high prevalence in the population in general, and is usually a consequence of one's life style. When asked more actively about the presence of sleepiness, patients tend to overrate the symptom, thus leading to an overestimation of the relationship between sleepiness and the medication. On the other hand, it is known that antihistamines do introduce a certain degree of sleepiness. This study did not have a control group, and it is therefore impossible to tell how much sleepiness derived from the medication itself and how much originated from patients overrating the symptom. However, most patients had mild symptoms that did not interfere with their productivity at work or adversely impact their social lives. Additionally, ^13^(8%) patients improved from sleepiness after the first week of treatment, indicating that the effects of rupatadine upon the central nervous system are mild, not very prevalent (+10%), and that they tend to improve with use as also shown in other studies^22^. No adverse cardiac events were reported in the study, an important addition to the safety of rupatadine, as arrhythmia cases have been described in association with other antihistamines as terfenadine.

All other adverse events related to the use of rupatadine had similar prevalence rates to those reported in other studies, including what was observed in the control groups of these studies, making it even harder to interpret the occurrence of such adverse events. However, for safety reasons the observed events should be seen as potentially connected to the medication.

## CONCLUSION

No control groups were defined in this study to compare rupatadine against other drugs or placebo. Thus, the assessment of efficacy is subject to other factors, such as the natural history of the disease or placebo effect.

Therefore, we may conclude that even in the Brazilian population rupatadine can effectively manage the signs and symptoms of moderate persistent allergic rhinitis, and that its effect lasted for the period of two weeks. We may also add that rupatadine in therapeutic dosages is safe and offers low side effect rates.
